# A Novel Potential Landmark for Intraoperative Estimation of Femoral Stem Anteversion: An Analysis of Computed Tomography Measurements

**DOI:** 10.3390/jcm15030945

**Published:** 2026-01-24

**Authors:** Ho Hyun Yun, Woo Seung Lee, Young Bae Kim, Jun Woo Park

**Affiliations:** Department of Orthopaedic Surgery, Veterans Healthcare Service Medical Center, Seoul 05368, Republic of Korea

**Keywords:** lesser trochanter, anterior lesser trochanter line, femoral anteversion, femoral stem anteversion, hip arthroplasty

## Abstract

**Background/Objectives:** Femoral anteversion (FA) is the angle between the femoral neck axis (FNA) and the posterior condylar axis (PCA). Surgeons generally estimate intraoperative femoral stem anteversion visually relative to the PCA, but this method can be challenging even for experienced surgeons. This study aimed to find an anatomical proximal landmark within a CT coordinate system for intraoperative estimation of femoral stem anteversion. **Methods:** Seventy patients were included. The anterior lesser trochanter line (ALTL) was defined as the line passing through two tangent points: one between the rounded part of the lesser trochanter and the medial edge of the anterior lesser trochanter cortex, and the other between the anterior cortex of the femur and the lateral edge of the anterior lesser trochanter cortex at the level of the lesser trochanter tip. The following angles were measured and analyzed: Angle 1 (angle between the FNA and the ALTL), Angle 2 (angle between the ALTL and the PCA), and Angle 3 (angle between the FNA and the PCA [FA]). **Results:** In all measurements, the inter-observer and intra-observer intraclass correlation coefficients exceeded 0.81. Angle 1 negatively correlated with Angle 2 (r = −0.79, *p* < 0.01), whereas Angle 1 positively correlated with Angle 3 (r = 0.70, *p* < 0.01). **Conclusions:** We found a consistent relationship between the ALTL and FA, and the ALTL may serve as a proximal landmark for intraoperative estimation of femoral stem anteversion during anterior or anterolateral approaches.

## 1. Introduction

Femoral anteversion (FA) is a significant contributor to hip joint function and is implicated in many joint pathologies [1]. Femoral stem anteversion is one of the most important factors influencing the outcome of total hip arthroplasty (THA) [2]. Improper positioning of the femoral stem anteversion can lead to persistent pain, decreased range of motion, excessive wear, dislocation, and implant failure due to impingement of the femoral neck on the rim of the acetabular cup [3,4,5,6].

FA is the angle between the femoral neck axis (FNA) and the posterior condylar axis (PCA) in the axial plane of the femur [7]. CT scans have been considered the gold standard for the FA measurement due to their high accuracy and reliability [7,8,9]. There are 2 main methods for measuring FA by CT data: the head–neck superimposition method [10] and the single-slice method [11]. Surgeons generally estimate intraoperative femoral stem anteversion by visually assessing its position relative to the PCA [1,12]. However, this method can be challenging even for experienced surgeons [1,12,13]. Several studies have explored proximal femoral anatomical landmarks for measuring femoral stem anteversion [1,12,13,14,15,16,17], but their utility for intraoperative estimation of femoral stem anteversion has not been clearly established.

The lesser trochanter, a bony projection on the femur, has been used in preoperative planning for hip arthroplasty [12,18,19]. The authors hypothesized that the position of the lesser trochanter can serve as a proximal landmark for intraoperative estimation of femoral stem anteversion.

This study introduces the anterior lesser trochanter line (ALTL) as a novel proximal anatomical landmark for intraoperative estimation of femoral stem anteversion. We evaluated its reliability via CT-based measurement, aiming to provide a simple and practical reference tool for surgeons.

## 2. Materials and Methods

### 2.1. Materials

**Figure 1:**
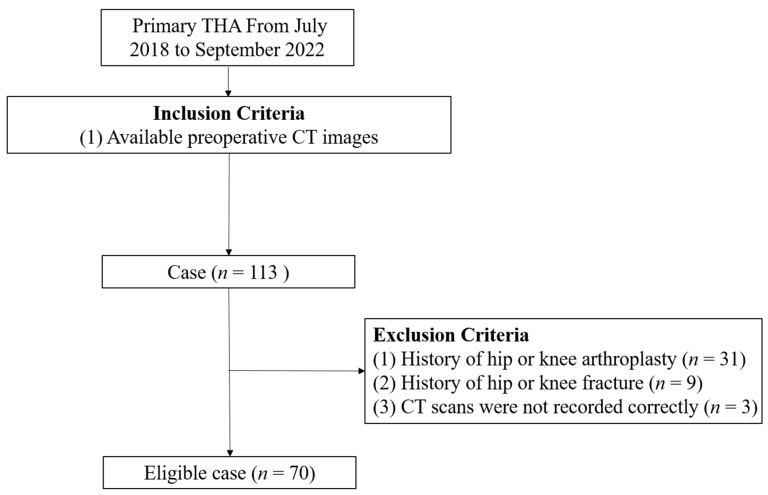
A flow chart illustrating patient enrollment.

This study retrospectively analyzed preoperative CT images scheduled for THA at a single institution between July 2018 and September 2022. CT scans were conducted as part of another study protocol [20]. Initially, 113 patients were selected for the study. However, patients were excluded if they had a history of hip or knee arthroplasty, hip or knee fracture, or insufficient CT scan data. After applying these exclusion criteria, CT images were taken from 70 patients/140 hips (50 men, 20 women; mean age, 66.0 ± 9.9 years; age range, 37–90 years) as the final study subjects (Figure 1). The preoperative diagnoses of hips included avascular osteonecrosis of the femoral head (*n* = 27), dysplastic hip (*n* = 17), osteoarthritis (*n* = 14), and post-traumatic osteoarthritis (*n* = 12).

### 2.2. CT Examination

CT images were acquired with the patient supine and symmetrically positioned (as confirmed by the scout view). The proximal CT scans extended from the fourth lumbar vertebra to 5 cm below the lesser trochanter, while the distal CT scans covered the distal femoral metaphysis to the femoral condyles. A support under the ankles stabilized the limb’s rotational position. All scans used a 2-mm slice thickness. All images were digitally acquired using a picture archiving and communication system (PACS). Assessments were performed on a 17-inch (1280 × 1024 pixels) liquid crystal display monitor using PACS software (ViewSTAR, version 5080, Infinitt, Seoul, Republic of Korea). The minimum detectable changes by the software were 0.1° in angle measurement and 0.1 mm in length measurement. To assess intra-observer and inter-observer measurement agreements, two sets of CT measurements with an interval of one month, of 30 randomly selected hips, were performed by three observers: a senior orthopedic surgeon (A, with 15 years of experience), a junior orthopedic surgeon (B, with 7 years of experience), and an orthopedic surgeon in training (C, with 3 years of experience).

### 2.3. CT Measurement

**Figure 2:**
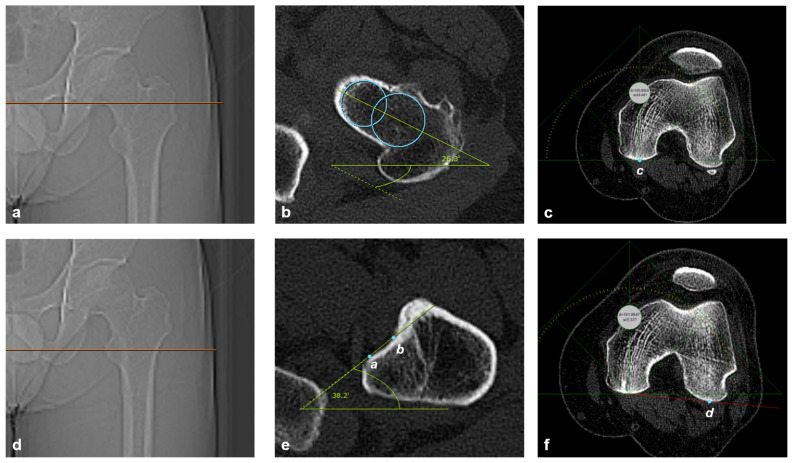
Measurement protocol for the FNA, ALT, and PCA angles. (**a**) Coronal scout image: The red line indicates the most proximal portion of the inferior neck distal to the femoral head. (**b**) Axial image: Measurement of the FNA angle. (**c**) Coronal scout image: The red line marks the level of the tip of the lesser trochanter. (**d**) Axial image: Measurement of the ALTL angle. (**e**,**f**) Axial image: Measurement of the PCA angle. *a*, Tangent point between the rounded part of the lesser trochanter and the medial edge of the anterior lesser trochanter cortex; *b*, Tangent point between the anterior femoral cortex and the lateral edge of the anterior lesser trochanter cortex; *c*, Lowest point of the medial femoral condyle; *d*, Lowest point of the lateral femoral condyle.

The following angles were measured: FNA, ALTL, and PCA. The single-slice method [11,12] was used for the FNA measurement. At the most proximal portion of the inferior neck distal to the femoral head (Figure 2a), the FNA was defined as the line passing through the midpoints of two circles: one at the medial and the other at the lateral part of the femoral neck (Figure 2b), and the FNA angle was defined as the angle formed between the FNA and the horizontal axis (Figure 2a,b). At the level of the tip of the lesser trochanter, which showed the largest width of the lesser trochanter among coronal images (Figure 2c), the ALTL was defined as the line passing through the two tangent points: one is between the rounded part of the lesser trochanter and the medial edge of the anterior lesser trochanter cortex, and the other is between the anterior cortex of the femur and the lateral edge of the anterior lesser trochanter cortex (Figure 2d). The ALTL angle was defined as the angle formed between ALTL and horizontal axis (Figure 2c,d). The PCA was defined as the line passing through the lowest portion of the medial and lateral femoral condyles [21]. First, place a screen ruler (MB-Ruler, open-source software; www.markusbader.de accessed on 15 December 2024) in the LMPFC (Figure 2e), and then place it in the level of LLPFC because the lowest portion of the medial posterior femoral condyle (LMPFC) and the lowest portion of the lateral femoral condyle (LLPFC) do not exist on the same plane. The PCA angle was defined as the angle formed between PCA and the horizontal axis (Figure 2f).

### 2.4. Data Analysis

The mathematical formulae used to determine the 3 angles (Angle 1, Angle 2, and Angle 3) were as follows:

* Angle 1 (angle between the FNA and the ALTL) = the FNA angle minus the ALTL angle.

* Angle 2 (angle between the ALTL and the PCA) = the ALTL angle minus the PCA angle.

* Angle 3 (angle between the FNA and the PCA [femoral anteversion]) = the FNA angle minus the PCA angle.

Angle values were positive for external rotation and negative for internal rotation relative to the horizontal axis.

### 2.5. Statistical Analysis

Standard descriptive statistics are reported as means ± standard deviations with 95% confidence intervals. Intraclass correlation coefficients (ICCs) assessed intra- and inter-observer reliability for the FNA, ALTL, and PCA angles. Normality was evaluated using the Kolmogorov–Smirnov test. For normally distributed data, a paired t-test was used to compare side-to-side angle differences; for non-normal data, the Wilcoxon signed-rank test was employed. Multiple linear regression was used to analyze side-to-side differences in all six angles (FNA, ALTL, PCA, Angles 1, 2, and 3) against demographic factors (gender, age, and diagnosis). Frequency distributions categorized absolute deviations from the mean (Δ < 5°, <10°, <15°, ≥15°). Pearson’s correlation coefficient was used to assess pairwise relationships among angles. G*Power 3.1.9.4 calculated the required sample size (70) for correlation analysis (α = 0.01, power = 0.90, effect size = 0.5). All analyses were performed in R (V 4.0.1; R Foundation) with statistical significance set at *p* < 0.01.

## 3. Results

In all measurements, the inter-observer and intra-observer ICC exceeded 0.81 (Table 1), indicating almost perfect agreement [22]. All angles were normally distributed (*p* = 0.29 to 0.85). The mean FNA angle was 18.4° ± 11.7, the mean ALTL angle was −39.8° ± 11.2, and the mean PCA angle was 4.1° ± 10.9° (Table 2). In side-to-side comparisons, Angle 3 (FA) showed a statistically significant difference (*p* < 0.01), whereas the ALTL angle did not (Table 3). For all six angles, >50% of values deviated from the mean by ≥5° (Table 4). Angle 1 negatively correlated with Angle 2 (r = −0.79, *p* < 0.01), while Angle 1 positively correlated with Angle 3 (r = 0.70, *p* < 0.01) (Table 5).

## 4. Discussion

Intraoperative estimation of femoral stem anteversion is predominantly accomplished through measuring the femoral stem’s position relative to the PCA. This is done by creating a horizontal plane by rotating the hip while holding the tibia straight upwards in the posterolateral approach or downwards in the anterior or anterolateral approach. However, this method may not be accurate due to the invisibility of the PCA in the surgical field, difficulty in maintaining consistency in horizontal leg position, and the effect of knee osteoarthritis [1,12,13,23]. Dorr et al. reported that surgeon-estimated femoral stem anteversion has poor accuracy [24]. Wines et al. concluded that intraoperative estimation of FA is of limited accuracy [7]. Uemura et al. have indicated that caution may be needed in using the PCA as a rotational reference for the femur [25]. Therefore, a proximal landmark in the surgical field would be beneficial in assessing intraoperative femoral stem anteversion. In this study, ICC results showed that the ALTL had almost perfect intra-observer and inter-observer agreement (Table 1), indicating excellent measurement repeatability and reproducibility. In this study, the ALTL was measured by three observers who had varying levels of orthopedic experience. The authors attribute these favorable results to two factors: (1) the relative simplicity of ALTL measurement, and (2) the enhanced accuracy enabled by image-processing software.

Our study has several limitations. First, the ALTL was evaluated only in CT scans, not in a clinical setting. Its clinical relevance requires further validation through additional research. Especially, it would be beneficial to study how soft tissue coverage might affect the intraoperative palpation or visualization of the “tangent points” defined on CT. Second, as an anterior structure, the ALTL may be challenging to visualize during posterolateral approaches. However, direct anterior [26,27] or anterolateral [28,29] approaches—having gained popularity over the past decade—could improve accessibility. Third, the ALTL was not compared to computer-assisted navigation [24]. Fourth, we could not use head–neck superimposition methods [10], which offer more precise FNA measurements than single-slice methods [11], due to the irregular femoral head morphology and the unavailability of 3D CT reconstruction. Nevertheless, the single-slice method remains sufficiently accurate for settings without a 3D image, provided scans are taken just below the femoral head [11].

The mean FA (14.3° ± 10.9) in this study was consistent with prior CT-based reports [8,9,30,31]. Previous studies highlighted FA variability within and across the populations [32,33,34], as well as individual asymmetry [35,36,37]. Aligning with these findings, >50% of individual Angle 1, Angle 2, and Angle 3 values in our study deviated from the mean value by <5° (Table 4). Additionally, our study found that Angle 3 showed significant side-to-side differences (*p* < 0.01), whereas Angle 2 and Angle 3 did not (Table 3). This supports Scorcelletti et al. [34] and Dimitriou et al. [37], who emphasized that native FA is patient-specific. Relying on contralateral femur FA during hip arthroplasty may not restore native FA, necessitating careful consideration in femoral stem positioning. A personalized approach to hip arthroplasty appears rational for optimizing patient outcomes.

To date, no standardized intraoperative method exists for accurately estimating femoral stem anteversion. Several studies [1,12,13,14,16,17] have explored proximal femoral anatomical landmarks for this purpose. Karnezis et al. [16] proposed using the first femoral broach (during initial canal preparation, before neck osteotomy) as a landmark to reproduce native FA in primary THA. However, this technique is limited in cases of abnormal FA. Virtual cut-surface landmarks, such as the midcortical line [14], T-line [13], and anterior/posterior femoral neck walls [1], have also been suggested. However, their accuracy depends on the height level of the neck osteotomy, leading to variability in FA estimation. The lesser trochanter’s position has been introduced as an anatomical landmark [12,17]. Unlu et al. defined the lesser trochanter axis/version on CT, reporting a mean version of 34.1° ± 3.0° (all values within 5° of the mean) [12]. Shon et al. confirmed a reliable correlation between the posterior lesser trochanter line and FA [17]. However, the lesser trochanter’s posterior and cephalad orientation makes it difficult to visualize the transverse plane, limiting the applicability of this landmark to approaches other than posterolateral. While computer-assisted navigation [38] and robotic arm-assisted technology [39] improve femoral stem anteversion accuracy, their high cost and limited accessibility restrict widespread use. This study addressed a practical surgical problem with a simple, anatomical solution. The ALTL is readily visible in the surgical field, unlike the PCA, which is not directly observable intraoperatively, making it a practical intraoperative landmark. In this study, ICC results showed that the ALTL had almost perfect intra-observer and inter-observer agreement (Table 1), indicating excellent measurement repeatability and reproducibility. Angle 1 (angle between the FNA and the ALTL) negatively correlated with Angle 2 (angle between the ALTL and the PCA) (r = −0.79, *p* < 0.01), whereas Angle 1 (angle between the FNA and the ALTL) positively correlated with Angle 3 (angle between the FNA and the PCA [femoral anteversion]) (r = 0.70, *p* < 0.01). These findings confirmed a consistent relationship between the ALTL and FA.

## 5. Conclusions

Given the challenges of visualizing the PCA during surgery and the high variability of native FA, the ALTL may serve as a proximal landmark for intraoperative estimation of femoral stem anteversion during anterior or anterolateral approaches. However, further studies are likely needed to validate its practical utility in clinical practice.

## Figures and Tables

**Table 1:** Intraclass correlation coefficients (ICCs) in the FNA angle, ALTL angle, and PCA angle.

Observer	FNA	ALTL	PCA
	Intra-observer ICC (95% confidence interval)		
A	0.92 (0.88–0.96)	0.95 (0.91–0.97)	0.93 (0.90–0.95)
B	0.93 (0.90–0.97)	0.97 (0.92–0.98)	0.95 (0.91–0.97)
C	0.91 (0.89–0.95)	0.98 (0.92–0.99)	0.96 (0.94–0.97)
	Inter-observer ICC (95% confidence interval)		
A-1 and B-1	0.91 (0.86–0.94)	0.95 (0.92–0.97)	0.93 (0.90–0.96)
A-1 and B-2	0.89 (0.84–0.91)	0.93 (0.90–0.96)	0.91 (0.86–0.94)
A-2 and B-1	0.90 (0.85–0.92)	0.95 (0.92–0.97)	0.92 (0.87–0.95)
A-2 and B-2	0.92 (0.88–0.95)	0.94 (0.91–0.97)	0.92 (0.88–0.95)
A-1 and C-1	0.89 (0.84–0.91)	0.95 (0.91–0.97)	0.93 (0.90–0.96)
A-1 and C-2	0.90 (0.85–0.92)	0.93 (0.90–0.96)	0.91 (0.86–0.94)
A-2 and C-1	0.91 (0.86–0.94)	0.95 (0.91–0.97)	0.92 (0.88–0.95)
A-2 and C-2	0.93 (0.90–0.96)	0.94 (0.91–0.95)	0.93 (0.90–0.96)
B-1 and C-1	0.90 (0.85–0.92)	0.95 (0.93–0.97)	0.91 (0.86–0.94)
B-1 and C-2	0.89 (0.85–0.92)	0.93 (0.89–0.95)	0.93 (0.90–0.96)
B-2 and C-1	0.90 (0.85–0.92)	0.94 (0.92–0.97)	0.91 (0.86–0.94)
B-2 and C-2	0.91 (0.86–0.94)	0.95 (0.92–0.97)	0.92 (0.88–0.95)

FNA, angle between the femoral neck axis and horizontal axis; ALTL, angle between the anterior lesser trochanter line and horizontal axis; PCA, angle between the posterior femoral condylar axis and horizontal axis.

**Table 2:** Summary of the six angles measured in both hips.

Parameter	Right Femur (*n* = 70) Mean ± SD (95% CI)	Left Femur (*n* = 70) Mean ± SD (95% CI)	Total (*n* = 140) Mean ± SD (95% CI)
FNA	19.7° ± 11.7 (16.9° to 22.5°)	17.0° ± 11.6 (14.2° to 19.7°)	18.4° ± 11.7 (16.4° to 20.3°)
ALTL	−39.8° ± 9.8 (−42.2° to −37.5°)	−39.8° ± 12.4 (−42.8° to −36.9°)	−39.8° ± 11.2 (−41.7° to −38.0°)
PCA	2.7° ± 11.3 (0.0° to 5.4°)	5.5° ± 10.4 (3.0° to 8.0°)	4.1° ± 10.9 (2.2° to 5.9°)
Angle 1	59.5° ± 12.3 (56.6° to 62.5°)	56.8° ± 12.3 (53.9° to 59.8°)	58.2° ± 12.3 (56.1° to 60.2°)
Angle 2	−42.5° ± 12.2 (−45.4° to −39.6°)	−45.3° ± 11.0 (−48.0° to −42.7°)	−43.9° ± 11.7 (−45.9° to −41.9°)
Angle 3	17.1° ± 11.3 (14.4° to 19.8°)	11.5° ± 9.7 (9.2° to 13.8°)	14.3° ± 10.9 (12.5° to 16.1°)

SD, standard deviation; CI, confidence interval; FNA, angle between the femoral neck axis and horizontal axis; ALTL, angle between the Anterior lesser trochanter line and horizontal axis; PCA, angle between the posterior femoral condylar axis and horizontal axis; Angle 1, angle calculated by subtracting the ALTL angle from the FNA angle; Angle 2, angle calculated by subtracting the PCA angle from the ALTL angle; Angle 3, angle calculated by subtracting the PCA angle from the FNA angle.

**Table 3:** Summary of the side-to-side difference in angles between the right and left sides.

Parameter	Side Difference Mean ± SD	95% CI of Side Difference	*p*-Value
FNA	2.7° ± 12.8	−0.3° to 5.8°	0.08
ALTL	0.0° ± 12.3	−2.9° to 3.0°	0.99
PCA	−2.8° ± 13.3	−6.0° to 0.3°	0.08
Angle 1	2.7° ± 10.7	0.2° to 5.3°	0.04
Angle 2	2.9° ± 11.1	0.2° to 5.5°	0.03
Angle 3	5.6° ± 9.5	3.3° to 7.9°	<0.01

SD, standard deviation; CI, confidence interval; FNA, angle between the femoral neck axis and horizontal axis; ALTL, angle between the Anterior lesser trochanter line and horizontal axis; PCA, angle between the posterior femoral condylar axis and horizontal axis; Angle 1, angle calculated by subtracting the ALTL angle from the FNA angle; Angle 2, angle calculated by subtracting the PCA angle from the ALTL angle; Angle 3, angle calculated by subtracting the PCA angle from the FNA angle.

**Table 4:** Distribution of the absolute differences between the mean value and individual values for each angle.

Parameter	Absolute Difference Number of the Hips (%)			
	<5°	<10°	<15°	≥15°
FNA	43 (30.7%)	76 (54.3%)	109 (77.9%)	31 (22.1%)
ATLT	56 (40.0%)	95 (67.9%)	119 (85.0%)	21 (15.0%)
PCA	49 (35.0%)	85 (60.7%)	118 (84.3%)	22 (15.7%)
Angle 1	34 (24.3%)	82 (58.6%)	112 (80.0%)	28 (20.0%)
Angle 2	53 (37.9%)	97 (69.3%)	111 (79.3%)	29 (20.7%)
Angle 3	48 (34.3%)	94 (67.1%)	115 (82.1%)	25 (17.9%)

FNA, angle between the femoral neck axis and horizontal axis; ALTL, angle between the Anterior lesser trochanter line and horizontal axis; PCA, angle between the posterior femoral condylar axis and horizontal axis; Angle 1, angle calculated by subtracting the ALTL angle from the FNA angle; Angle 2, angle calculated by subtracting the PCA angle from the ALTL angle; Angle 3, angle calculated by subtracting the PCA angle from the FNA angle.

**Table 5:** Correlation results among angles.

Parameter	FNA	ALTL	PCA	Angle 1	Angle 2	Angle 3
FNA	1	0.62 *	0.74 *	0.77 *	−0.11	0.73 *
ALTL	0.62 *	1	0.64 *	−0.71 *	0.74 *	0.71 *
PCA	0.74 *	0.64 *	1	0.11	−0.72 *	−0.63 *
angle1	0.77 *	−0.71 *	0.11	1	−0.79 *	0.70 *
angle2	−0.11	0.74 *	−0.72 *	−0.79 *	1	0.60 *
angle3	0.73 *	0.01 *	−0.63 *	0.70 *	0.60 *	1

FNA, angle between the femoral neck axis and horizontal axis; ALTL, angle between the Anterior lesser trochanter line and horizontal axis; PCA, angle between the posterior femoral condylar axis and horizontal axis; Angle 1, angle calculated by subtracting the ALTL angle from the FNA angle; Angle 2, angle calculated by subtracting the PCA angle from the ALTL angle; Angle 3, angle calculated by subtracting the PCA angle from the FNA angle. * *p* < 0.01.

## Data Availability

The original contributions presented in this study are included in the article. Further inquiries can be directed to the corresponding author(s).
